# Self-assembly mechanisms of wheat gluten peptides: Modulating interfacial behavior and foaming properties

**DOI:** 10.1016/j.fochx.2026.103486

**Published:** 2026-01-03

**Authors:** Jiabao Cao, Guangqi Fan, Baoxin Lu, Zhigang Xiao, Guang Xin

**Affiliations:** a College of Food Science, Shenyang Agricultural University, Shenyang, China; b College of Food Science, Heilongjiang Bayi Agricultural University, Daqing, China; c College of Food Science, Bohai University, Jinzhou, China

**Keywords:** Polypeptide hydrogel, Self-assembly, Wheat gluten protein, Foam stability, Interfacial properties

## Abstract

Foam performance plays a critical role in food systems by influencing texture and stability. This study investigates how the self-assembly of wheat gluten protein peptides (WGPs) affects foaming behavior and interfacial properties. Self-assembled WGP gel nanoparticles (WGPM-NPs, 3–10 kDa) produced interfacial layers with higher interfacial expansion viscoelastic modulus (E) and elastic sub-modulus (E_d_), resulting in enhanced foaming capacity (128.3 ± 22.3 %) and foam stability (39.1 ± 3.3 %). The interfacial layers stabilized by WGPM-NPs also exhibited increased composite modulus and strain-hardening behavior during expansion and compression, forming a highly elastic, solid-like two-dimensional gel interface that combines rigidity and flexibility. These characteristics contributed to improved long-term foam stability. Overall, the findings demonstrate that leveraging WGP self-assembly is an effective strategy to improve foam performance and regulate interfacial behavior, offering new insights into the application of self-assembled peptide gel nanoparticles in functional food systems.

## Highlights

• Molecular changes influenced the air–water interfacial behavior of the system.
• The interfacial layer stabilized by WGPM-NPs exhibited an enhanced foaming capacity.
• This layer also demonstrated a solid-like elastic behavior and a greater stability.
• Self-assembly tuning enhances the foam performance and the interfacial behavior

## Graphical abstract

**Figure:**
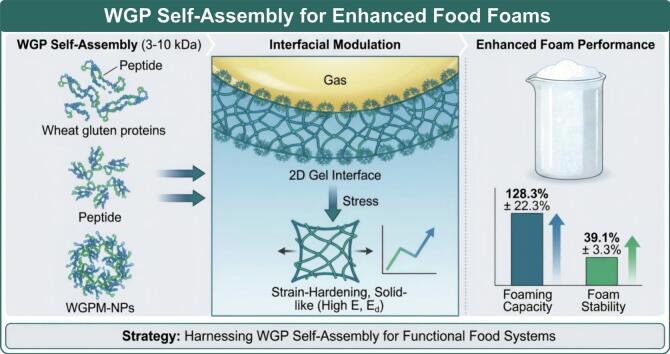

## 1 Introduction

Foams are unstable colloidal systems vital to the food industry, particularly affecting food texture and taste through their structure and stability. As a result, the use of foams in the food industry has gained significant attention in recent years (Abioye et al., 2024; Amagliani et al., 2021; Wu et al., 2022; Zhang et al., 2024). Proteins, acting as surfactants, have been shown to stabilize foam structures by forming thin films at the gas-liquid interface and reducing surface tension (Bertsch et al., 2021; Yang et al., 2021; Zhu et al., 2020). However, food protein-based surfactants face certain limitations at the air/water interface. Due to their larger size, their migration to the air/water interface is slow, and their interfacial activity is weak, impacting foam stability (Wang et al., 2024). Studies on colloidal particles from plant proteins like zein, gluten, kafirin, and soy protein revealed that small-molecule surfactants are often needed to enhance their stability at the air/water interface, forming stable Pickering foams (Sarkar & Dickinson, 2020; Fameau, Guzmán, Ritacco and Saint-Jalmes, 2023a, Fameau, Guzmán, Ritacco and Saint-Jalmes, 2023b; Nirmal, 2024). Additionally, several methods have been proposed to modify the air/water interface. For instance, regulating protein structures and functions can improve their performance at the air/water interface, while combining proteins with other amphiphilic components can further tune interfacial rheology and foam stability (Amagliani et al., 2021; Zhang et al., 2024; Zhu et al., 2021). Moreover, recent studies indicate that using nanotechnology or bottom-up self-assembly to create protein or peptide-based particles can effectively boost interfacial activity and foam performance (Li, Liu, et al., 2023; Zhu et al., 2024). With advances in biotechnology and nanoscience, studying the behavior and performance of biomaterials at the nanoscale has gained increasing significance. In particular, self-assembling food proteins and peptides can form fibrils, gels, and nanoparticles with tunable structures and functions (Liu et al., 2023; Li, Xiao, et al., 2023; Zhu et al., 2024).

Wheat gluten protein (WG), a renewable and widely available protein, has potential applications in food, medicine, and materials science due to its ability to self-assemble into peptide gel nanoparticles (Valencia et al., 2019; Tomadoni et al., 2020; Yu et al., 2022; Li, Xiao, et al., 2023; Cao, Fan, & Lu, 2025; Liu et al., 2023). WG mainly consists of glutenin and gliadin, which aggregate into a three-dimensional network via non-covalent interactions, imparting wheat dough with its unique rheological properties (Feng et al., 2021). However, this also renders WG insoluble in water, limiting its industrial applications. WG is rich in sulfur-containing proteins (e.g., α-, β-, γ-gliadin), which contain abundant cysteine residues that readily form disulfide bonds with other proteins, enhancing hydrogel stability and elasticity (Liu et al., 2023; Cao, Fan, & Lu, 2025). Additionally, the hydrophilic central domain and hydrophobic terminals of high-molecular-weight glutenin subunits (HMW-GS) facilitate intermolecular interactions, which are essential for forming hydrogels with strong mechanical properties and elasticity (Rodriguez & Hemar, 2020). Despite these promising attributes, the inherent insolubility of WG poses a barrier to its direct use as a surfactant in aqueous-based foam systems, where rapid interfacial adsorption is crucial. To address this, enzymatic hydrolysis or mild processing can derive WGP from WG, unlocking its latent amphiphilicity for nanoscale self-assembly while preserving its renewable abundance as an agro-waste-derived resource. During foam preparation, the hydrophilic groups of surfactants disperse in the aqueous phase, while hydrophobic groups interact with air. These molecules quickly adsorb, unfold, and rearrange, forming a stable viscoelastic air/water interfacial film (Mohamed, 2023). Therefore, the complex and heterogeneous nature of WG, along with its amphiphilic properties, facilitates self-assembly, enabling WGP gel nanoparticles to act as particle-like surfactants.

Previous studies indicate that heat- and acid-treated WGP, as well as gluten-derived peptide systems, can exhibit excellent colloidal stability and expansive rheological properties at the air/water interface, suggesting their potential in food systems requiring stable foam structures (Fuentes-Prado & Martínez-Padilla, 2014; Wouters et al., 2016; Wouters, Fierens, et al., 2017; Bertsch et al., 2021; Liu et al., 2023). Recent work has further shown that WGP-based systems can be engineered into self-assembled nanoparticles with good loading capacity and interfacial activity for hydrophobic bioactives, highlighting the versatility of gluten-derived nanostructures in soft-matter applications (Zhang et al., 2024; Cao, Fan, Wang, & Lu, 2025). Together with earlier studies on wheat gluten hydrolysates and their interfacial behavior, these findings point to WGP as a promising building block for sustainable foaming agents (Wouters et al., 2018).

In our previous study, we elucidated the self-assembly mechanisms of WGP, identifying key interactions responsible for their formation (Cao et al., 2025a). However, to our knowledge, there are no reports on the air/water interfacial properties of self-assembled hydrogel nanoparticles of WGP as particle-like surfactants. The goal of this study is to explore the interfacial behavior and foaming properties of self-assembled WGP hydrogels. Building upon previous insights into the self-assembly mechanism, we now analyze the functional characteristics of these hydrogels at the air/water interface. Rheological performance, interfacial expansion rheology, and Lissajous curves were employed to examine their interfacial properties, while the foaming ability and stability of the nanoparticles were assessed macroscopically. This study seeks to deepen the understanding of how molecular structure influences interfacial behavior and foam performance. By applying straightforward processing techniques, we aim to demonstrate the potential of WGP as natural foaming agents, contributing to their use in the food foaming industry and supporting the development of more diverse and functional food formulations.

## 2 Materials and methods

### 2.1 Materials

Vital wheat gluten was provided by Baoqi Agricultural Science and Technology Development Co., Ltd. (Mudanjiang City, China). Trypsin (200,000 U/g) was obtained from porcine pancreas, and purchased from Sigma-Aldrich (Shanghai, China). All aqueous solutions were prepared using deionized water (Milli-Q water). All other chemicals and solvents were of analytical grade.

### 2.2 Preparation of wheat gluten polypeptide hydrogel

#### 2.2.1 Extraction of wheat gluten protein

WG was extracted from vital wheat gluten, which contains approximately 75 % protein, along with wheat starch, cellulose, and lipids. Despite the high protein content, lipid and polysaccharide impurities in vital wheat gluten could affect test results, leading to inaccurate data. Therefore, a triple alkali-soluble acid precipitation method was used to extract WG (Cao et al., 2024).

The WG was dispersed in deionized water, and the pH was adjusted to 12 with 1 mol/L NaOH solution, followed by 2 h of stirring with an overhead stirrer. The protein solution was then centrifuged at 10,000*g* for 20 min to remove starch and impurities. The supernatant was collected, and the pH adjusted to 4.0 with 1 mol/L HCl. The precipitated crude protein was collected by centrifuging again at 10,000*g* for 20 min. The precipitate was washed three times with deionized water to remove salts, then freeze-dried to obtain the WG sample, with a protein content of 89.27 %.

#### 2.2.2 Wheat gluten proteolysis

Following the methodology described by Cao et al. (2024). A WG sample was weighed and placed into a 1000 mL isothermal enzyme reactor. The sample was uniformly dispersed into a 5 % (*w*/*v*) suspension by adding deionized water under continuous stirring. The isothermal tank was set to the optimal protease reaction temperature of 40 °C. The initial pH of the solution was adjusted to 8.0 using 1 mol/L sodium hydroxide or hydrochloric acid, and trypsin was added at a 1:100 enzyme-substrate ratio to hydrolyze the WG. During the reaction, an alkali solution was continuously added to keep the pH of the hydrolysate within the optimal range. Samples were collected at 0, 10, 30, 60, and 120 min, and the degree of WG hydrolysis was determined using the pH-stat method. After enzyme inactivation, the sample was cooled to room temperature, centrifuged at 10,000*g* for 15 min, and the supernatant was separated and frozen for later use. The degree of hydrolysis (DH) of WG was determined using the pH-stat method. DH was calculated according to the following equation:

(1) $$\mathrm{DH}\left(\%\right)=\frac{B\times N_{b}}{M_{p}\times h_{\mathrm{tot}}}\times 100$$

Where: *B* is the volume (L) of NaOH solution consumed during hydrolysis, *N*_*b*_ is the molar concentration (mol/L) of the NaOH solution, *M*_*p*_ is the mass (g) of protein in the reaction system, and ℎ_tot_ = 7.59 mmol/g.

#### 2.2.3 Ultrafiltration fractionation and preparation of self-assembled nanoparticles

Following the methodology described by Cao et al. (2024). At room temperature, the RNF-0460 spiral wound membrane separation system (Sida Membrane Technology Co., Ltd., Xiamen, China) was operated at an ultrafiltration pressure of 0.1 MPa. Ultrafiltration membranes with various molecular weight cut-offs were attached to the system. The enzymatic hydrolysate (WG-H) was fractionated into four groups based on molecular weight: >10 kDa (WGP-L), 10–3 kDa (WGP-M), <3 kDa (WGP—S), and unfractionated WG-H. Each fraction was freeze-dried.

Freeze-dried WG hydrolysate (WG-H) and peptide powders (WGP-L, WGP-M, WGP—S) were dispersed in deionized water (pH 7.0) at a concentration of 15 % (*w*/*v*). After 2 min of ultrasonic agitation at room temperature, the mixtures were allowed to stand for 10 min, forming four types of self-assembled peptide hydrogels (WG-H, WGPL-NPs, WGPM-NPs, WGPS-NPs). The freeze-dried hydrogels were subsequently used to analyze foam characteristics and air/water interfacial behavior.

#### 2.2.4 Scanning Electron microscopy (SEM)

For scanning electron microscopy (SEM) analysis, a Quattro S SEM (Thermo Fisher, Germany) was employed to examine the morphology of the samples. The samples were initially frozen in liquid nitrogen at −80 °C and immediately crushed to preserve structural integrity. Subsequently, the samples were vacuum freeze-dried using a freeze dryer (Huaxing Biotechnology Co., Ltd., Beijing, China). After freeze-drying, the samples were mounted onto conductive adhesive, and their fractured surfaces were sputter-coated with gold using an Oxford Quorum SC7620 sputter coater (UK) for 45 s at 10 mA. Finally, the morphology of the samples was observed under the SEM at an acceleration voltage of 3 kV.

### 2.3 Foam performance

#### 2.3.1 Foaming ability and foam stability

The prepared foam was sheared continuously at 100,000 rpm for 120 s using an Ultra-Turrax T 25 (IKA, Germany) at room temperature. The resulting foam was immediately transferred to a graduated cylinder for observation (Li et al., 2022). Foaming capacity (FC) and foam stability (FS) were calculated using the following equations:

(1) $$\mathrm{FC}\left(\%\right)=\frac{V_{t}-V_{0}}{V_{0}}$$

(2) $$\mathrm{FS}\left(\%\right)=\frac{V_{a}-V_{0}}{V_{t}-V_{0}}$$

Where: *V*_t_ (mL) is the initial volume of the foam, *V*_0_ (mL) is the initial volume of the liquid, and *V*_a_ (mL) is the volume of the foam recorded after 60 min.

#### 2.3.2 Observation of foam morphology

The foam's microscopic structure, stored for varying durations, was observed using a fluorescence microscope (BX 53, Olympus Corporation, Japan). Prior to observation, a small portion of labeled foam was placed on a concave slide and covered with a coverslip.

#### 2.3.3 Foam rheological properties

The rheological properties of all foam systems were measured at 25 °C using a rotational rheometer (AR 2000ex, TA Instruments, USA) equipped with a plate-plate geometry (plate diameter: 40 mm). Freshly prepared foam was placed on the base, with a fixed gap of 2 mm between the upper plate and the base. After 30 s of standing, strain sweeps were performed at 6.283 rad/s across a strain range of 0.01 % to 10 %, recording changes in storage modulus (G') and loss modulus (G") to determine the linear viscoelastic region. Steady-state flow experiments were conducted by measuring the shear rate from 0.1 to 100 s^−1^. Steady-state shear experiments were conducted at a shear rate of 0.05 s^−1^ for 1000 s, recording shear stress (Pa) over time (t). Frequency sweep tests were conducted on freshly prepared foam at 0.05 % strain across a frequency range of 0.1 to 10 rad/s. Subsequently, frequency sweep data were fitted using power-law equations:

(3) $$G^{’}=K^{\prime }∙\omega^{n’}$$

(4) $$G^{"}=K^{"}∙\omega^{n\prime \prime }$$

where *G'* and *G"* are the storage modulus and loss modulus, respectively, ω is the angular frequency (rad/s), and *K*′, *K″*, *n*’, and *n*” are the viscoelastic coefficients of the system.

#### 2.3.4 Foam Micro-rheological analysis

The dynamic behavior of complex fluid systems, such as foam, was analyzed using an optical micro-rheometer (Formulaction, France) based on the Diffusing Wave Spectroscopy (DWS) principle at 25 °C. Freshly prepared foam was carefully added into a flat-bottomed cylindrical glass tube (8 cm height, 2 cm diameter), avoiding large air gaps and excessive shear during loading. The sample was allowed to stand for 30 s before measurement. A camera recorded statistical parameters from the light reflected by the sample, including the Elasticity Index (EI), Fluidity Index (FI), Solid-Liquid Balance (SLB), and Macroscopic Viscosity Index (MVI). The data were analyzed using DWS software, recording the Mean Squared Displacement (MSD) of particles as a function of time lag, from which the micro-rheological indices were obtained.

### 2.4 Air/water interfacial behavior analysis

An interfacial rheometer (Tracker, Teclis, France) was used to measure the interfacial tension and expansion rheological properties of self-assembled nanoparticles at the gas–liquid interface at 25 °C. All samples were prepared as 25 mL solutions with a 1 % (*w*/*v*) concentration and placed in quartz cuvettes. A suspended bubble was created on the liquid surface using a gas-tight syringe, and the cuvette was sealed with plastic wrap to minimize evaporation effects. After bubble formation, the system was equilibrated for 30 s before data collection. A CCD camera recorded real-time data such as bubble area and volume, and the interfacial tension was determined from the bubble profile via axisymmetric drop-shape analysis (ADSA). For interfacial expansion (dilatational) rheology, the bubble interfacial area was subjected to small-amplitude periodic area oscillations, and the corresponding interfacial response was analyzed to obtain the expansion rheological parameters.

#### 2.4.1 Dynamic surface pressure measurement

Bubble image analysis was conducted using system software to calculate surface tension during adsorption. Surface tension (γ) was calculated using the Young-Laplace equation. Surface pressure (π) was calculated using the following formula:

(5) $$\pi =\gamma_{\left(0\right)}-\gamma_{\left(t\right)}$$

where *γ*_(0)_ is the interfacial tension of pure water at 25 °C (72.8 mN/m), and *γ*_(t)_ is the interfacial tension of the test sample solution at adsorption time *t*.

#### 2.4.2 Amplitude sweep

Previous studies often overlooked the effect of interfacial deformation amplitude on rheological properties, assuming that measurements were confined to the linear viscoelastic region without experimental evidence. To investigate the response of self-assembled gluten protein peptide nanoparticles to deformation, we followed the method outlined by Zhan et al. (2020). All experiments were conducted at 25 °C. After reaching equilibrium in dynamic surface tension, amplitude sweeps were performed at a constant frequency of 0.1 Hz, with amplitudes ranging from 1.5 % to 30 %.

#### 2.4.3 Interfacial expansion rheological properties

Rheological parameters for interfacial expansion were determined by inducing periodic sinusoidal expansions and compressions at a specified frequency (f) and amplitude (dA/A) using a computer-controlled system. Following Zhan et al. (2020), the frequency (f) was set to 0.1 Hz with a 10-s period, and the amplitude (dA/A) was kept at 10 % to remain within the linear viscoelastic range. The linear interfacial expansion viscoelastic modulus (E) is defined as the ratio of the relative change in interfacial area (dA/A) to the change in interfacial tension (dσ) (Lucassen & Van Den Tempel, 1972).

(6) $$\sigma =\sigma_{0}\sin\left(\omega \theta +\delta \right)$$

(7) $$A=A_{0}\sin\omega \theta$$

(8) $$E=\frac{\mathrm{d\sigma}}{\mathrm{dA}/A}=\frac{\mathrm{d\pi}}{d\ln A}=E_{d}+iE_{v}$$

Here, *σ*_*0*_ represents the initial surface tension, *A*_*0*_ the initial surface area, and *δ* the phase angle between stress and strain. The real part (*E*_*d*_ = |E| cos δ) corresponds to the elastic modulus, reflecting the elastic behavior of the viscoelastic interface. The imaginary part (*E*_v_ = |E| sin δ) corresponds to the viscous modulus, reflecting the viscous behavior of the viscoelastic interface. The interfacial expansion viscoelastic modulus evaluates the membrane's resistance to deformation, where |E| represents the total resistance of the material to elastic and viscous deformation.

#### 2.4.4 Frequency sweep

To evaluate the rheological properties of the interface formed by self-assembled nanoparticles under varying frequency conditions and reveal their internal structure and dynamic behavior, experiments were conducted at 25 °C. After dynamic surface tension reached equilibrium, a frequency sweep was conducted at a constant amplitude of 5 % across a frequency range of 0.005 to 0.1 Hz. The slope of the modulus-frequency curve was determined using double logarithmic linear fitting.

#### 2.4.5 Lissajous curve

Lissajous curves, following the method outlined by Van Kempen et al. (2013a), were generated to further examine the interfacial expansion rheological properties. These curves illustrate the relationship between surface pressure (π = γ-γ_0_) and surface deformation (δA/A_0_, where δA = A-A_0_). Here, γ and A represent the real-time surface tension and interfacial area during the amplitude sweep, while γ_0_ and A_0_ denote the initial, undeformed surface tension and area.

Four key parameters can be extracted from the Lissajous curves through quantitative analysis: maximum expansion strain modulus (*E*_*L,E*_), maximum compression strain modulus (*E*_*L,C*_), minimum expansion strain modulus (*E*_*M,E*_), and minimum compression strain modulus (*E*_*M,C*_). Finally, the interfacial strain stiffness ratio (S) is calculated from these parameters to quantify the nonlinearity of the interface:

(9) $$S_{\mathrm{ext}}=\frac{E_{L,E}-E_{M,E}}{E_{L,E}}$$

(10) $$S_{\mathrm{com}}=\frac{E_{L,C}-E_{M,C}}{E_{L,C}}$$

The interface's compression and expansion responses are distinguished as follows: S = 0 indicates a linear elastic response, S > 0 signifies strain hardening during expansion, and S < 0 denotes strain softening during the cycle.

### 2.5 Statistical analysis

All experiments were conducted in triplicate under identical conditions, with data presented as mean ± standard deviation (SD). Statistical analyses were performed using Origin 2021 and SPSS Statistics 24.0. One-way analysis of variance (ANOVA) was applied to detect statistical differences between means, with significance set at *P* < 0.05.

## 3 Results and discussion

### 3.1 Characterization of self-assembled hydrogels from wheat gluten protein peptides

**Fig. 1:**
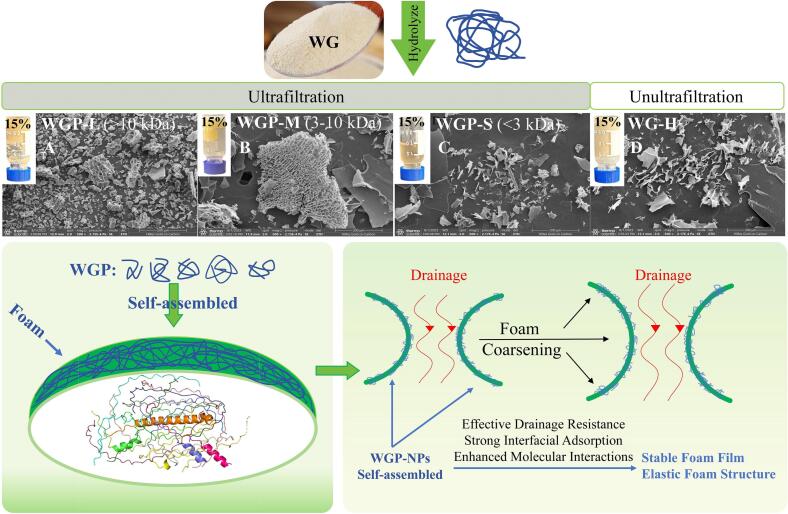
Morphology of self-assembled WGP gel and adsorption mechanism of WGP-NPs at the foam interface.

In our previous research, we confirmed that WGP can form self-supporting hydrogels (Fig. 1**A** and **B**) through self-assembly in an aqueous phase, driven by electrostatic and hydrophobic interactions (Cao et al., 2024). Therefore, this study explores how the molecular mechanisms of self-assembled gluten protein peptide nanoparticles (WG-H, WGPL-NPs, WGPM-NPs, WGPS-NPs) affect foam properties and interfacial adsorption behavior (Fig. 1).

### 3.2 Foam performance analysis

#### 3.2.1 Analysis of the foaming capacity, foam stability, and foam morphology

**Fig. 2:**
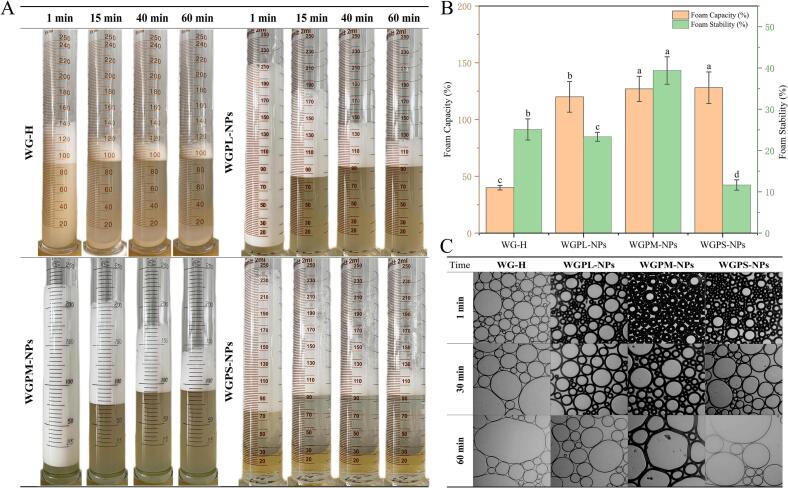
Macro image of foam (A), foam capacity and stability (B), and microscopic foam image (C). Values with different letters are statistically different (*p* < 0.05).

The foam properties of surfactants are crucial factors during the preparation of aerated foods (e.g., cakes and ice cream), and the behavior of each different self-assembled peptide structure at the air/water interface is considered to play a key role in the peptide chain aggregation process. These considerations are important when studying peptide chain aggregation during the foam fractionation process (Wang et al., 2018). Thus, the foam performances of different nanoparticles were observed during a storage period of 1–60 min (Fig. 2A). As the molecular weight of the samples decreased, the foaming capacity (FC) and foam stability (FS) initially increased and then decreased, confirming that the effect of ultrafiltration fractionation on the samples was not entirely positively correlated, and that the self-assembly mechanisms of peptides with different molecular weights also play important roles. As presented in Fig. 2B, the untreated WG-H exhibits the lowest FC (41.5 ± 3.5 %), while the WGPS-NPs species exhibits the lowest FS (11.6 ± 1.26 %), and the WGPM-NPs exhibit the highest FC (128.3 ± 22.3 %) and FS (39.1 ± 3.3 %). These results are similar to the findings of Van der Vent et al. (2002), who showed that whey protein and casein peptides exhibit their optimal foam formation abilities in the molecular weight range of 3–5 kDa. Studies have shown that the foaming properties of protein systems are mainly influenced by factors such as the molecular size, surface hydrophobicity, and surface charge. A higher surface hydrophobicity results in a higher foaming capacity (Li et al., 2021). However, in this study, it was found that the WGPM-NPs possessed the lowest surface hydrophobicity but the highest FC and FS values. It was therefore speculated that this may be related to the influence of the interface self-assembled peptide gel network on the foam performance. Additionally, a study by Sheng et al. (2020) found that the co-assembly of hydrophobic particles and hydrophilic substances at the interface can lead to the formation of ultra-stable foams. This also demonstrates that the interactions of the peptides at the interface are crucial for determining the foam properties.

The foam micrographs (Fig. 2C) show that as the storage time increased, all four samples exhibited a reduction in foam quantity and an increase in foam coarsening. However, in the foam prepared using the WGPM-NPs, the foam coarsening phenomenon was alleviated compared with those observed in the WG-H, WGPL-NPs, and WGPS-NPs groups. Notably, the size of the bubbles plays a decisive role in the stability of the a foam system. Further observations revealed that the foam size distribution in the WGPM-NPs was more uniform, and the foam arrangement was tighter. The WG-H, WGPL-NPs, and WGPS-NPs specimens exhibited larger foam volumes at the same storage time, and the presence of larger bubbles increased the likelihood of system instability. Further in-depth analysis of the WGPM-NPs foam structure revealed thicker bubble walls and tighter structures during the 60 min storage period, leading to a larger contact area and a smaller contact angle. These results indicate that the WGPM-NPs form thicker and more elastic interfacial films at the air/water interface, increasing the foaming performance and stability of the solution (Wouters et al., 2020). Alzobaidi et al. (2021) demonstrated that an increase in the interfacial elastic modulus prevented the expansion, contraction, movement, and coalescence of bubbles. When the interactions between the molecules at the interface increase, the viscoelasticity of the interfacial film also increases, which enhances the macroscopic foam stability of the system (Da et al., 2021). In fact, the FCs and FSs of surfactants are complex properties influenced by many factors, such as the molecular size, surface charge, hydrophobicity, adsorption, and rearrangement ability at the gas–liquid interface, in addition to the diffusion rate of gas between the interfacial films (Ma et al., 2020). Thus, the interfacial behavior of the nanoparticles on the micro-interfacial scale was further explored to establish a connection to the macroscopic foam properties.

#### 3.2.2 Foam rheological performance analysis

**Fig. 3:**
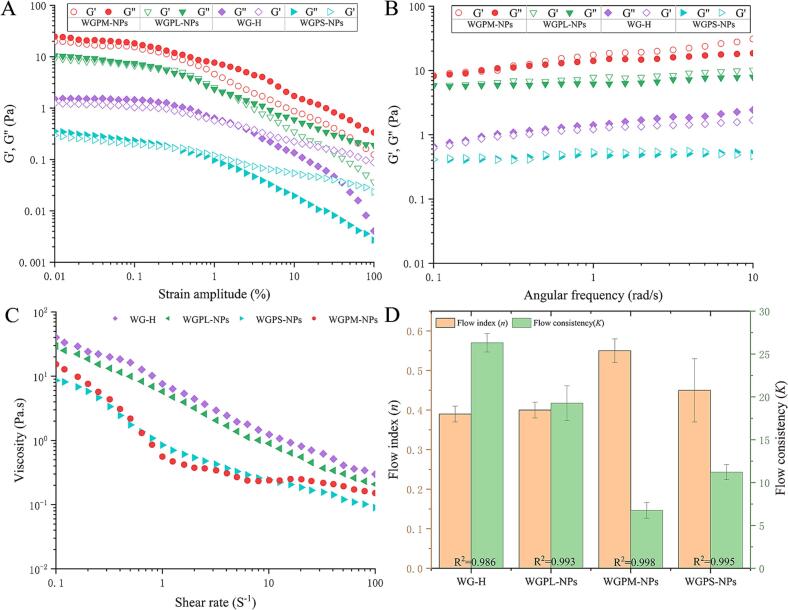
Viscoelastic moduli (*G*' and *G*") as a function of strain for foam generated by WG-H, WGPL-NPs, WGPM-NPs, and WGPS-NPs (A). Elastic/viscous moduli (*G*' and *G*") as a function of frequency for foam generated by WG-H, WGPL-NPs, WGPM-NPs, and WGPS-NPs (B). Apparent viscosity as a function of shear rate for foam generated by WG-H, WGPL-NPs, WGPM-NPs, and WGPS-NPs (C). Viscosity parameters of foams generated by WG-H, WGPL-NPs, WGPM-NPs, and WGPS-NPs (D).

Foams are highly complex systems, wherein the bubble size, deformation sensitivity, and mechanical properties affect the final rheological performance. From a macroscopic perspective, the rheological behavior of a foam is crucial for determining its physical stability. Thus, the viscoelastic properties of the prepared foams were evaluated using strain and frequency sweep tests. It is well known that the storage modulus (G') and loss modulus (G′′) characterize the contributions of the elasticity and viscosity to a material. As shown in Fig. 3A, the foam prepared using the WGPM-NPs exhibited the highest G' value, thereby confirming that this foam exhibits a superior stability (Alzobaidi et al., 2021). Further analysis revealed that in the linear viscoelastic region (0.01–0.1 %), the elastic modulus in the foam system approached the viscous modulus, and the G' and G" values of all foam samples rapidly decreased when the strain exceeded 0.1 %, indicating a nonlinear viscoelastic response (Vishal., 2021). The reason for this phenomenon may be that, under high strain conditions, the foam exhibits a liquid-like behavior. More specifically, the bubbles break under a high shear stress to form smaller bubbles and align in the flow direction, exhibiting a pseudo-liquid behavior. Fig. 3B shows the frequency sweep data for this foam, wherein it can be seen that in a frequency range of 0.1–10 Hz, the G' values of all foam samples (with the exception of WG-H) are slightly higher than their corresponding G" values, corresponding to a weak gel-like network structure (Sheng et al., 2020). Notably, as the frequency was increased, the gap between G' and G" widened for the WGPM-NPs foam. This phenomenon may be due to WGPM-NPs self-assembly at the interface generating a stronger gel network structure, which is also supported by previous research results (Zhu et al., 2020). To further evaluate the types of structures present in the various foams, power-law equation (G = *Kωⁿ*) fitting frequency sweep analysis was performed for the frequency dependences of G' and G". As shown in Table 1, the foam prepared using the WGPM-NPs possessed a larger *K* value and a smaller *n* value than the other foams prepared. This may be because the presence of the self-assembled peptide gel network enhances the solid-like behavior of the foam (smaller *n* value) and increases the elastic strength of the foam system (larger *K* value) (Clarke et al., 2019).

**Table 1:** Parameters of the power-law model. Power Law Consistency Index (*K′*), Power Law Flow Index (*n*’), Coefficient of Determination (R^2^), Complex Power Law Consistency Index (*K*″), Complex Power Law Flow Index (*n*”), for the fit of *K*″ and *n*”(R^2^). The letters indicate significant differences (*p* < 0.05).

Sample	*K′* (Pa·s^n’^)	*n’*	*R*^*2*^	*K″* (Pa·s^n’^)	*n”*	*R*^*2*^
WGPM-NPs	8.84 ± 0.37^a^	0.18 ± 0.04^c^	0.992	7.95 ± 1.13^a^	0.13 ± 0.01^c^	0.989
WGPL-NPs	5.97 ± 0.26^b^	0.21 ± 0.02^b^	0.991	5.72 ± 1.09^b^	0.17 ± 0.02^b^	0.987
WG-H	0.48 ± 0.05^c^	0.49 ± 0.03^a^	0.996	0.43 ± 0.02^c^	0.45 ± 0.13^a^	0.993
WGPS-NPs	0.41 ± 0.07^c^	0.08 ± 0.01^d^	0.979	0.44 ± 0.02^c^	0.04 ± 0.01^d^	0.965

Subsequently, the relationship between the apparent viscosity and the shear rate was systematically investigated for the WG-H, WGPL-NPs, WGPM-NPs, and WGPS-NPs foams. As presented in Fig. 3C, the apparent viscosities of all samples decreased with an increasing shear rate, exhibiting shear-thinning behavior similar to those of pseudoplastic fluids, wherein the viscosity and shear rate have an inverse functional relationship (Jabarkhyl et al., 2020). At the same time, all samples showed a low flow behavior index (*n* < 1, Fig. 3D), which also indicates that they can be classed as pseudoplastic fluids. However, it is noteworthy that the foam prepared using the WGPM-NPs exhibited a thickening behavior at high shear rates. Notably, viscosity changes caused by variations in the shear rate are crucial for subsequent foaming processes because they provide details regarding the polymer brittleness under certain shear conditions, which is important in determining the foam stability (Kabziński et al., 2019). This phenomenon may therefore be due to the self-assembly mechanism of the WGPM-NPs, which increases the interactions between nanoparticles (Santini et al., 2019).

#### 3.2.3 Foam Micro–rheological performance analysis

**Fig. 4:**
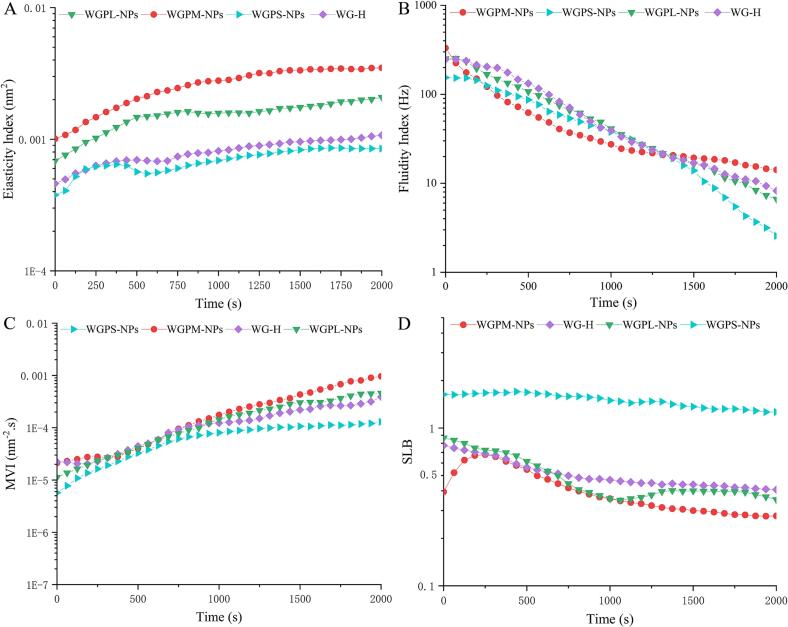
Micro-rheological properties of foam stabilized by WG-H, WGPL-NPs, WGPM-NPs, and WGPS-NPs: Elasticity index (A); Fluidity index (B); Macroscopic viscosity index (C); and Solid-liquid balance (D).

Macroscopic rheological methods are important for studying the mechanical properties of food colloids and have significant practical implications (Xu et al., 2021). However, macroscopic shear rheology methods can generalize the properties of materials at the macroscopic level but cannot provide detailed information on local changes in the microstructure (Milani & Golkar, 2019). Therefore, to further study the rheological properties of the WG-H, WGPL-NPs, WGPM-NPs, and WGPS-NPs foams, micro-rheological techniques were used to evaluate their structures and dynamic characteristics at the microscopic level. Fig. 4 shows the relevant parameters of the foam systems, with Fig. 4A presenting details regarding the elasticity index (EI), which defines the elastic strength of a sample. The EI value of the WGPM-NPs foam was found to be significantly higher than those of the other samples, which is consistent with the experimental shear rheology results (Jain et al., 2021). In addition, the flowability index (FI) represents the mobility of active substances in a foam system. As shown in Fig. 4B, the FI values of all samples decreased with time, indicating that movement of the active substances was restricted. It can be seen that the foam stabilized using the WGPM-NPs exhibited a slightly higher FI over time than the other samples, indicating that WGPM-NPs can effectively stabilize the foam bubbles (Nabizadeh & Jamali, 2021). Furthermore, the macroscopic viscosity indices (MVIs) of all foam samples (Fig. 4C) were found to increase over time, indicating that the overall macroscopic viscosity increased, thereby leading to enhanced stabilities for all foam systems. Moreover, the WGPM-NPs foam exhibited higher MVI values over a long period of time, indicating that WGPM-NPs stabilization led to a lower movement speed, which imparts the foam system with a good stability. This is consistent with the results of a previous study (Minami et al., 2019). The solid–liquid balance (SLB, Fig. 4D) is defined as the ratio of solid-to-liquid-like behavior of a sample. For a SLB of 0.5, equal liquid-like and solid-like behaviors are observed. However, when 0.5 < SLB < 1, liquid-like behaviors dominate, and when 0 < SLB < 0.5, solid-like behaviors dominate (i.e., gel behavior). Fig. 4D shows that the SLB value of the foam stabilized by the WGPS-NPs exceeded 0.5, indicating that it was dominated by a liquid-like behavior. In contrast, for the foams stabilized by the WG-H, WGPL-NPs, and WGPM-NPs species, the SLB values were > 0.5 at *t* < 700 s, indicating that their stable foam systems were primarily dominated by liquid-like behaviors in the initial stage. At *t* > 700 s, the SLBs of the WG-H and WGPL-NPs foams approached 0.5, while that of the WGPM-NPs-stabilized foam gradually decreased over time and was significantly lower than the corresponding values of the other samples, indicating that solid-like behavior dominated. This further illustrates the positive effect of the self-assembled gel network in the WGPM-NPs foam system on the foam stability, which is consistent with the experimental shear rheology results (Park & Rogers, 2020).

### 3.3 Air–water interfacial behavior analysis

In this section, an intrinsic connection is established between the interfacial behaviors of the WG-H, WGPL-NPs, WGPM-NPs, and WGPS-NPs systems and their FC and FS values by evaluating the interfacial tension and interfacial expansion rheological properties of the self-assembled gel nanoparticle systems.

#### 3.3.1 Interfacial expansion rheology analysis

**Fig. 5:**
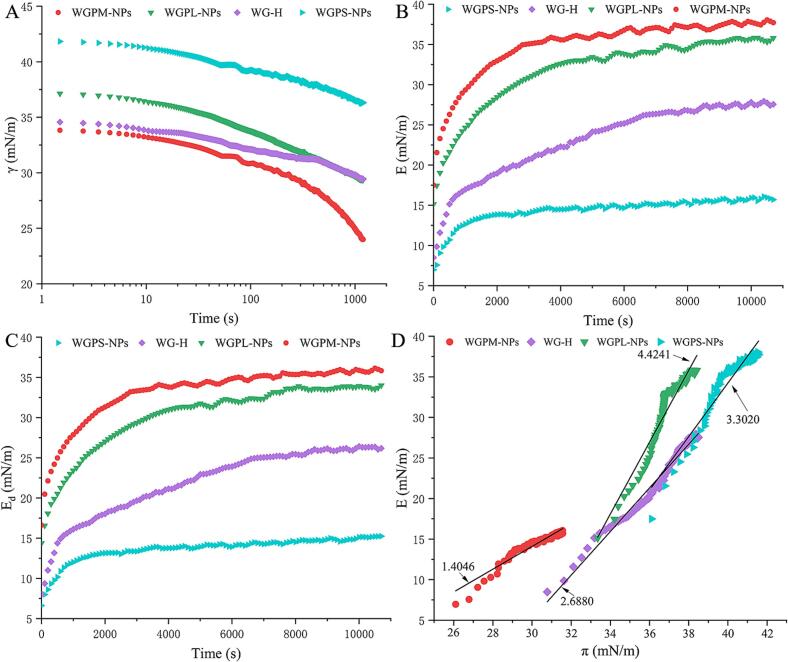
Surface tension (*γ*) of WG-H, WGPL-NPs, WGPM-NPs, and WGPS-NPs systems on the air/water interface as a function of adsorption time (A). Surface dilatational modulus (B) and elastic modulus (C) of the air/water interface stabilized by WG-H, WGPL-NPs, WGPM-NPs, and WGPS-NPs as a function of adsorption time. Surface dilatational modulus (E) plotted as a function of surface pressure (π) (D).

As shown in Fig. 5A, the interfacial tensions (γ) of the WG-H, WGPL-NPs, WGPM-NPs, and WGPS-NPs systems changed over time (t), decreasing gradually due to the adsorption of active substances onto the interface. Notably, the initial interfacial tension of the WGPM-NPs system was significantly lower than those of the other samples. Additionally, the rate of decrease in the interfacial tension was significantly higher for the WGPM-NPs system, which also demonstrates that the WGPM-NPs can rapidly adsorb onto the gas–liquid interface (Suleymani et al., 2020). According to previous studies (3.2. Foam Performance Analysis), the FC and FS values of the WGPM-NPs are superior to those of the WG-H, WGPL-NPs, and WGPS-NPs. Therefore, by comparing the changes in the FC/FS and in the surface tension, it was found that the ability of WGPM-NPs to reduce the surface tension is positively correlated with the foaming capacity (Yekeen et al., 2019). This is also consistent with previous studies that related the foaming capacity to the interfacial adsorption kinetics (Xu et al., 2021).

Fig. 5**B** and **C** show the changes in the interfacial expansion viscoelastic modulus (E) and the elastic modulus (E_d_) over time for the different samples. It can be seen that the E and E_d_ values of all samples show a significant increasing trend over time, which was attributed to their adsorption at the interface. In addition, the E and E_d_ values of the WG-H, WGPL-NPs, and WGPS-NPs samples were lower than those of the WGPM-NPs sample, indicating that the stable interfacial film generated by the WGPM-NPs exhibits a superior elastic behavior (Alzobaidi et al., 2021). These results indicate that the peptide segments enhance the connectivity of the gel network at the interface when connected in a noncovalent self-assembled manner. Therefore, the enhancement in the WGPM-NPs foam stability may be related to an increase in the interfacial expansion viscoelastic modulus (Zhou et al., 2019).

To further elucidate the effect of nanoparticle self-assembly on the interfacial rheology, Fig. 5D plots the relationship between E and surface pressure (π). All slopes were > 1 (N.B., the slope of an ideal adsorption curve is equal to 1), indicating that the gas-water biphasic interfacial films exhibited nonideal gas properties, in addition to the ability to form gel-like interfacial structures (Ni et al., 2020). Further analysis revealed that the WGPM-NPs system gave the closest slope to the ideal value of 1 (i.e., 1.4046). Previous studies have shown that moderate slope values (i.e., slightly greater than 1) can achieve a good balance between the interfacial film stability and the foam performance. In contrast, high slope values can result in overly rigid interfacial films, whereas excessively low slope values can lead to insufficient interfacial film strengths (Bertsch & Fischer, 2019). This may be due to the face that the self-assembly of nanoparticles in the interfacial layer imparts a good flexibility to the interfacial structure. The rigid adsorption layer becomes flexible, enhancing the ability of the interfacial film to respond to external deformation and effectively preventing foam coalescence and rupture, thereby providing superior FS and FC values (Da et al., 2021). This validates our previous findings (3.2. Foam Performance Analysis).

#### 3.3.2 Amplitude sweep and frequency sweep analysis

**Fig. 6:**
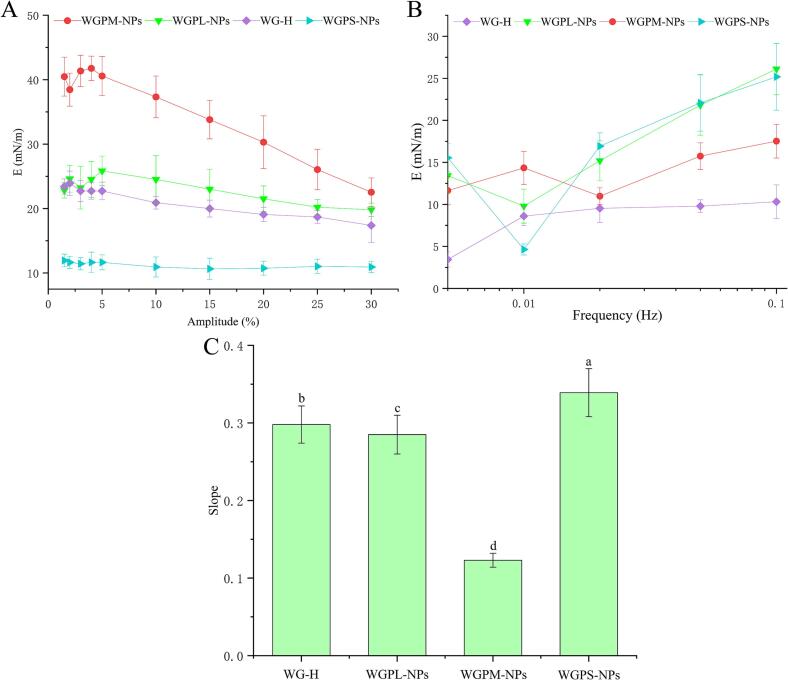
Relationship between surface dilatational modulus and amplitude of the air/water interface stabilized by WG-H, WGPL-NPs, WGPM-NPs, and WGPS-NPs (A). Relationship between surface dilatational modulus and frequency of the air/water interface stabilized by WG-H, WGPL-NPs, WGPM-NPs, and WGPS-NPs (B). Slope value of the modulus versus frequency curve, fitted by double logarithmic equation, for WG-H, WGPL-NPs, WGPM-NPs, and WGPS-NPs (C).

Amplitude and frequency sweep analyses were performed to gain a deeper understanding of the interfacial mechanical properties and microstructures of the stable interfaces of the self-assembled WGP nanoparticles. As shown in Fig. 6A, in the low amplitude range of 1.5–5 %, the E values of all samples showed irregular changes, exhibiting characteristics corresponding to a linear viscoelastic region. This phenomenon may have occurred because the diffusion rate of the nanoparticles moving from the bulk phase to the interface was lower than the deformation rate of the interface. When the amplitude range was 5–30 %, the E values of all samples decreased with an increasing amplitude, wherein the WGPM-NPs system exhibited the most significant effect. This indicates that a high amplitude affects the microstructure of the interface and the gradual formation of an interfacial film structure. It was therefore inferred that the rheological response of the interface upon variation in the amplitude was influenced by the self-assembly of peptide chains, i.e., the interface response was affected by the self-assembly interactions of the peptide chains adsorbed at the interface (Feng et al., 2021).

The frequency sweep results of the stable interfaces of all samples are shown in Fig. 6B, wherein it can be seen that the E values increased with an increasing frequency, demonstrating an obvious frequency dependence, and indicating that all samples exhibited a good elastic response behavior (Chen & Zou, 2019). Using the Lucassen-van den Tempel model (Lucassenand & Van Den Tempel, 1972), a logarithmic linear fitting curve analysis was performed on the frequency sweep results. The slopes of the logarithmic linear fitting curves of E with respect to the frequency are shown in Fig. 6C. For a slope value of 0.5, the diffusion rate of the active molecules between the solution and the interface dominated the elastic expansion behavior. Notably, frequency changes mainly affect the diffusion dynamics of the active molecules, resulting in a specific frequency dependence of the surface expansion modulus. In this case, the interface response is primarily regulated by the diffusion of surfactant molecules between the interface and the solution (Ghosh et al., 2019). When the slope approaches 0.0, the interface exhibits a fully elastic response, wherein the elastic modulus is mainly determined by the interactions between the surfactant molecules adsorbed at the interface, rather than by the diffusion process. In this case, the interface exhibited stable elastic properties with almost no viscous loss when the frequency changed, indicating that the elastic modulus of the interface was primarily governed by the interactions between the surfactant molecules. Notably, the slope of the WGPM-NPs curve was determined to be 0.123, which is close to zero, indicating that the interface has a high elastic response. Thus, the elastic modulus is mainly determined by the interactions between the surfactant molecules adsorbed at the interface, i.e., the interface response is influenced by the self-assembly interactions of the peptide chains adsorbed at the interface (Wychowaniec et al., 2020).

#### 3.3.3 Lissajous curve analysis

**Fig. 7:**
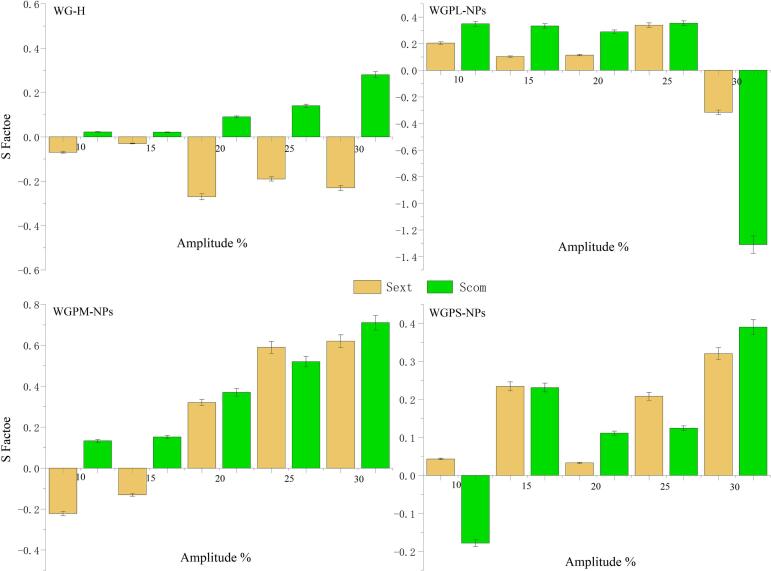
S factor for amplitude scanning of the air/water interface stabilized by WG-H, WGP-NPs, WGPM-NPs, and WGPS-NPs during extension and compression.

In recent years, the use of Lissajous curves to represent the relationship between the stress or surface pressure and the deformation has sparked scientific interest in terms of the nonlinear response behavior of the interfacial rheology. Thus, to further analyze the interfacial behaviors of the samples, Lissajous curves were used to comprehensively characterize the relationship between the surface pressure and the degree of deformation, with the aim of establishing a potential connection between the interfacial rheological response behavior and the interfacial microstructure. Notably, the Lissajous curves for purely elastic behavior are completely linear, whereas perfectly circular Lissajous curves indicate a purely viscous behavior at the interface. A nonlinear behavior at the interface leads to asymmetric Lissajous curves, which may soften upon expansion or harden upon compression. Therefore, to quantify the degree of the nonlinear response, the strain stiffness ratio (S) was introduced to quantitatively analyze the asymmetry during the expansion (S_ext_) and compression (S_com_) deformations (Fig. 7) (Giménez-Ribes et al., 2019).

**Fig. 8:**
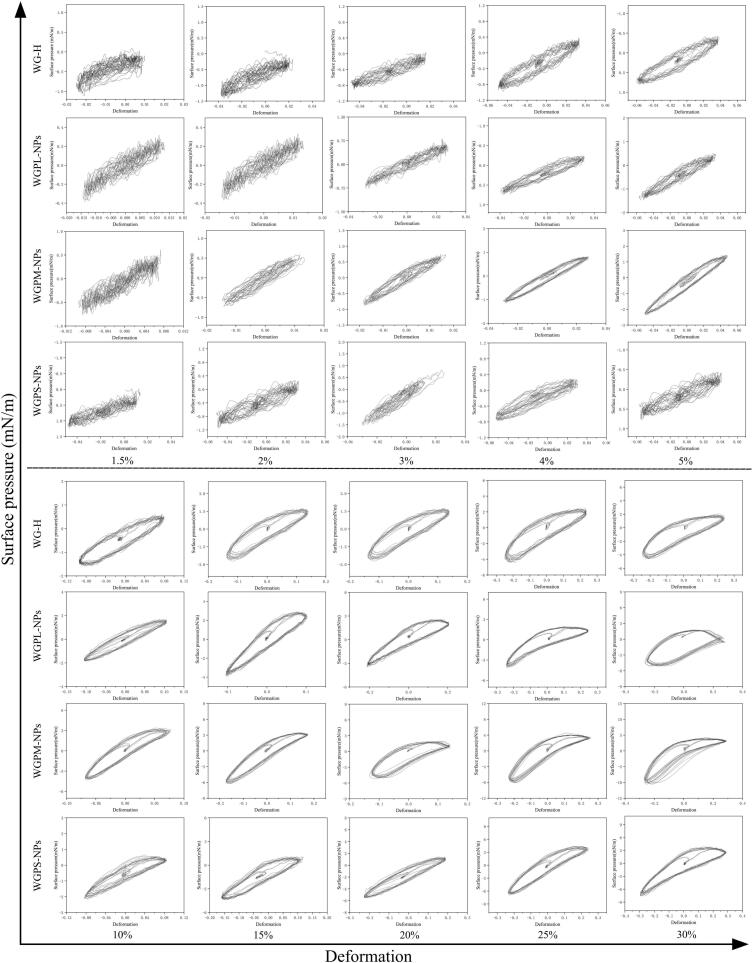
Lissajous curves of air/water interfaces stabilized by WG-H, WGPL-NPs, WGPM-NPs, and WGPS-NPs.

From the Lissajous curves in Fig. 8, it can be clearly observed that for an amplitude range of 1.5–5 %, all samples exhibited significant noise (disordered curves), which may be due to the bubble deformation rate of the tested samples being significantly higher than the interfacial adsorption rate (Javadi et al., 2021). For an amplitude range of 10–15 %, the Lissajous curves of all samples showed no obvious asymmetry, and the S values were small and close to 0 (Fig. 7), indicating that the interface mainly exhibited an elastic response behavior. At higher amplitudes (20–30 %), the Lissajous curves of all the samples showed significant asymmetry, indicating the nonlinear response behavior of the interfacial layer. Notably, the S values of the WGPM-NPs system at high amplitudes (20–30 %) were > 0 (Fig. 7), indicating a transition from a strain-softening to a strain-hardening interfacial response behavior, with the interface exhibiting a highly elastic two-dimensional gel structure (Goudoulas & Germann, 2019). This result indicates that the interactions of WGPs at the interface under the driving force of self-assembly increase the elasticity of the interfacial film. These results further confirm that WGPM-NPs can form an interfacial layer structure with a strong viscoelasticity, thereby imparting a good foam stability to the system (Li et al., 2022).

## 4 Conclusions

In summary, the self-assembly behavior between peptides in the WGPM-NPs plays a crucial role in enhancing foam performance and interfacial adsorption. WGPM-NPs showed superior macroscopic foam performance with FC and FS. Micrographs revealed thicker bubble walls, a more compact structure, a larger contact area, and a smaller contact angle between foams. Furthermore, self-assembled nanoparticles at the air/water interface exhibited lower initial and final surface tension values. The stable interfacial layer displayed higher interfacial expansion viscoelastic modulus (E) and elastic modulus (E_d_), correlating with enhanced foam stability for WGPM-NPs. Besides, interfacial expansion rheology and Lissajous curve results indicated that WGPM-NPs formed interfaces with high expansion complex modulus, demonstrating solid-like elastic behavior characterized by strain hardening during both expansion and compression. WGPM-NPs combine rigidity and flexibility, enabling rapid response to external deformations and ensuring long-term macroscopic stability of the foam system. The relationship between self-assembled nanoparticle mechanisms, foam performance, and interfacial behavior has been clarified, with the aim of developing natural food additives that offer enhanced foaming performance and stability for the food industry.

## CRediT authorship contribution statement

**Jiabao Cao:** Writing – review & editing, Software, Methodology, Formal analysis, Conceptualization. **Guangqi Fan:** Writing – review & editing, Validation, Methodology, Conceptualization. **Baoxin Lu:** Writing – review & editing, Validation, Supervision, Methodology, Conceptualization. **Zhigang Xiao:** Writing – review & editing, Formal analysis. **Guang Xin:** Writing – review & editing, Supervision, Methodology.

## Declaration of competing interest

The authors declare that they have no known competing financial interests or personal relationships that could have appeared to influence the work reported in this paper.

## Data Availability

Data will be made available on request.
